# Systemic retinoids for treatment of recalcitrant IgA pemphigus

**DOI:** 10.1186/s13023-018-0899-y

**Published:** 2018-09-18

**Authors:** Franziska Schauer, Johannes Steffen Kern, Dimitra Kiritsi

**Affiliations:** 1 Department of Dermatology, Medical Center- University of Freiburg, Faculty of Medicine, University of Freiburg, Hauptstr. 7, 79104 Freiburg, Germany; 2 Royal Melbourne Hospital, Parkville and Box Hill Hospital - Monash University Eastern Health Clinical School, Box Hill, VIC Australia

**Keywords:** Autoimmune skin blistering, Alitretoin, Acitretin, Desmosomes, Desmocollin

## Abstract

IgA pemphigus is an exceedingly rare autoimmune blistering disorder, caused by IgA autoantibodies against desmosomal proteins. No treatment option has been found to be universally effective. The disease is often recalcitrant to oral steroids and immunosuppressants. Here, we describe the use of systemic retinoids for the treatment of recalcitrant IgA pemphigus in 3 cases. Although the use of acitretin has been reported before, we present for the first time the positive effects of alitretinoin in treatment of 2 patients with IgA pemphigus. Besides hyperlipoproteinaemia requiring use of hypolipidemic agents in one case, alitretinoin was well-tolerated and has generally a more favorable side effect spectrum than immunosuppressants.

**Electronic supplementary material:**

The online version of this article (10.1186/s13023-018-0899-y) contains supplementary material, which is available to authorized users.

## Introduction

IgA pemphigus is an autoimmune blistering disorder, caused by IgA autoantibodies against keratinocyte cell surface antigens, desmocollins 1–3 and sometimes desmogleins 1 and 3. Based on clinical and histopathological characteristics it can be subdivided into subcorneal dermatosis type (SPD) and intraepidermal neutrophilic type (IEN) [1, 2] The patients present with flaccid pustules solely or on scaly erythematous plaques, often arranged in an annular or circinate pattern [3, 4], which are commonly itchy and located on the trunk. No treatment option has been found to be universally effective. The disease is often recalcitrant to local and/ or oral steroids and immunosuppressants [5]. Given the extreme rarity of the disease no controlled treatment trials exist or are likely to be conducted. We describe the novel use of alitretinoin in two out of three of our IgA pemphigus patients as a steroid-sparing agent.

## Patients and methods

**Fig. 1:**
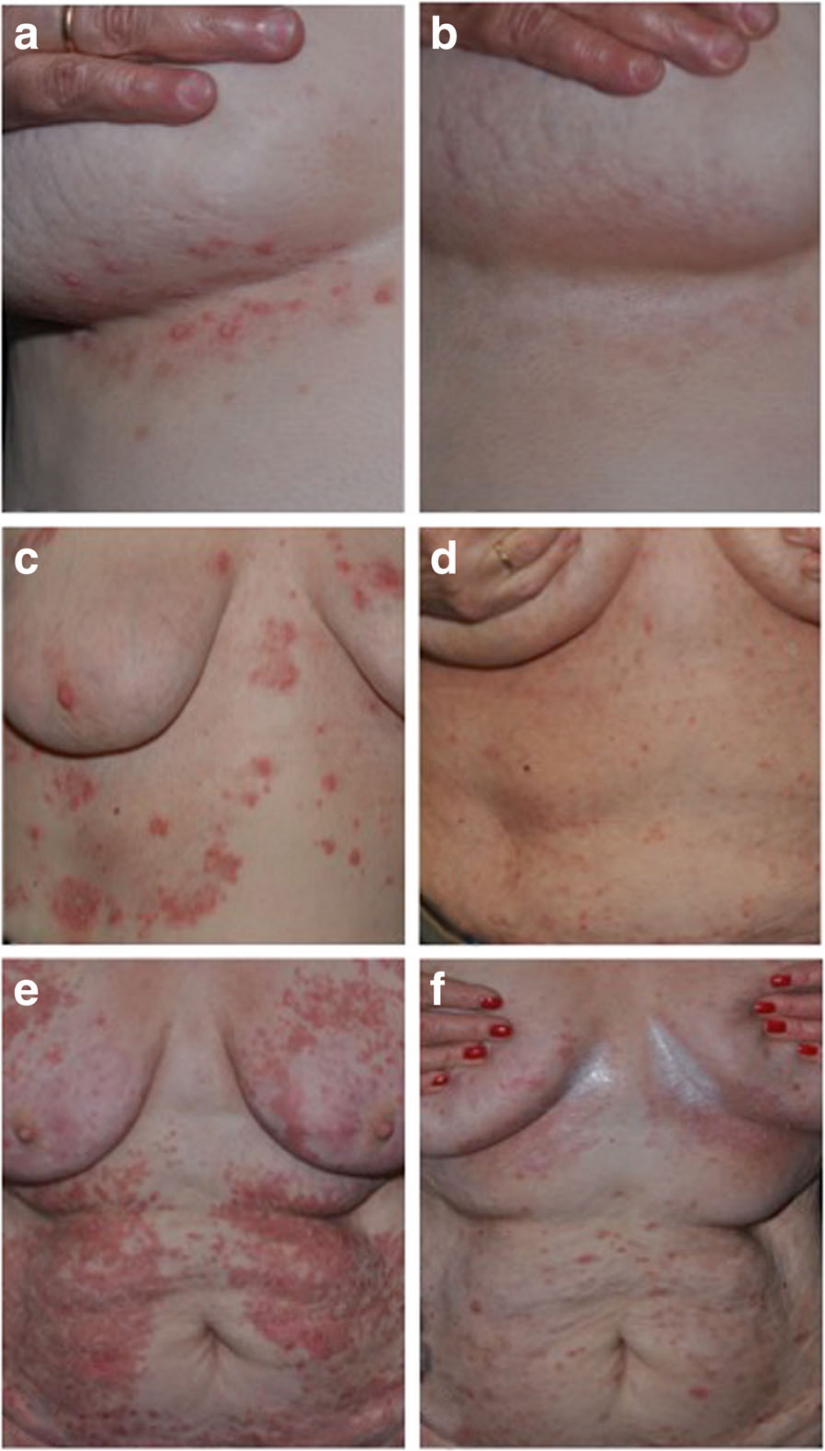
All three patients are shown before and 2–3 months after treatment initiation with systemic retinoids. Patient 1 has flaccid vesicles within her submammary folds (**a**), with complete resolution around three months after initiation of alitretinoin (**b**). Patient 2 presented with confluent pustules on erythematous plaques (**c**), which improved significantly within three months after initiation of alitretinoin (**d**). The third patient had confluent erythematous papules and pustules on her trunk (**e**) with partial amelioration two months after initiation of acitretin at a dosage of 10 mg per day (**f**)

Over the last 10 years we cared for three female patients with IgA pemphigus, with disease presenting around the age of 60 (Fig. 1). The diagnostic analyses performed are summarized in Table 1 and Additional file 1: Figure S1. Two of them have an underlying monoclonal gammopathy of undetermined significance (MGUS). Bence Jones light chains are negative. In the other case an underlying haematological condition or other malignancy was excluded. Interestingly, all three patients had a recalcitrant disease with at least four immunosuppressive or immunomodulatory drugs given, resulting in unsatisfactory response and/ or accompanied by intolerable side effects (Table 1). Besides local steroids the following treatments were used: dapsone, azathioprine, mycophenolate mofetil, colchicine, prednisolone, methotrexate, anakinra, cyclosporine and cyclophosphamide. In all three patients we initiated treatment with acitretin at dosage of 10–30 mg, which has been reported before to be effective in a few cases [6].

**Table 1:** Summary of the patient history with clinical presentation and drug history, as well as the diagnostic analyses performed in all 3 patients reported here

No.	Sex	Age^a^	Clinical presentation	DIF	IIF ME	IB	Gammopathy	Previous treatment, dosage and duration^b^	Systemic prednisolone dosage	Retinoid dosage	Side effects
1	F	63	Solid flaccid blisters primarily in intertriginous areas (subcorneal pustular dermatosis type)	Subcorneal IgA IC	IC IgA	DSC3	Monoclonal IgGκ, BJ proteins negative	Azathioprine (2.5 mg/kg) (15 mo) colchicine (1.5 mg/d) (4 mo) acitretin (0.3 mg/kg) (3 mo) anakinra (1 g) (once) methotrexate (15 mg/we) (3 mo) ^c^	5 mg	Alitretinoin 30 mg/d (since 3 yrs.)	Acitretin: hair loss, extreme dryness of skin and mucosa, hyperlipoproteinaemia, reduction of visual acuity Alitretinoin: hyperlipoproteinaemia
2	F	57	Multiple, annular sterile pustules on erythematous plaques on the trunk (subcorneal pustular dermatosis type)	Subcorneal IgA IC	IC IgA	DSC3	Not found	Azathioprine (1.5 mg/kg) (3 mo, hepatopathy MMF 2 g (6 mo) MMF 2 g + dapsone (1 mg/kg) (6 mo) colchicine (3 mg/d) (2 yrs.) acitretin (0.5 mg/kg) (4 mo)	7.5/ 10 mg	Alitretinoin 30 mg/d (since 2 yrs.)	Acitretin: hair loss, dizziness, dryness of mucosa None under alitretinoin
3	F	57	Disseminated single and confluent sterile pustules on erythematous plaques on the trunk and les on the extremities (subcorneal pustular dermatosis type)	Subcorneal IgA IC	IC IgA	n.a.	Monoclonal IgAλ, BJ proteins negative	Cyclophosphamide + fortecortin (cum. 11 mg) (11 months) azathioprine (2 mg/kg) (3 mo) thalidomide (100 mg/d) (3 mo) ciclosporine (~ 2 mg/kg) (3.5 yrs.) ciclosporine (~ 2 mg/kg) + acitretin (0.5 mg/d) (3 mo) acitretin (0.5 mg/d) + dapsone (1.5 mg/kg) (6.5 yrs)	0 mg	Acitretin 10 mg/d (since 6 yrs.)	Acitretin: discrete hyperlipoproteinaemia

^a^Age at time of diagnosis; *BJ*, Bence Jones light chains, *cum*, cumulative dosage, *DSC* desmocollin, *IC* intercellular, *ME* monkey esophagus, *MMF* mycophenolate mofetil, *mo* months, *κ* kappa, *λ* lambda; *n.a.* not available, *yrs*. years

^b^The previous treatments are presented in chronological order

^c^Patient 1 has glucose-6-phosphate-dehydrogenase deficiency, thus dapsone was not a treatment option

## Results and discussion

The treatment with the retinoid acitretin in all three patients resulted in a satisfactory, at least partial disease response. In two out of three cases, however, side effects (dizziness, hair loss and severe mucosal xerosis) occurred, prompting switch to the retinoid alitretinoin, which was given at a dosage of 30 mg daily. Alitretinoin is a novel systemic, endogenous retinoid acting as a pan-agonist for the nuclear retinoid receptors retinoic acid receptor (RAR) and retinoid-X-receptor (RXR). It is the first systemic treatment to be approved in the EU for patients with severe chronic hand eczema unresponsive to potent topical corticosteroids. It has also been used in pilot studies for other chronic inflammatory skin disorders [7]. Besides hyperlipoproteinaemia requiring use of hypolipidemic agents in one of our patients – one of the most commonly reported side-effect of alitretinoin [8] – the drug was well-tolerated and has a favourable side effect spectrum compared to immunosuppressants. This is specifically important in light of the increased risk for malignancies, observed in patients with IgA pemphigus [2, 9].

Our observations suggest that due to their antiinflammatory and antiproliferative functions, systemic retinoids and especially alitretinoin represent an excellent treatment option for IgA pemphigus, an exceedingly rare autoimmune blistering skin disease, which is commonly recalcitrant to different treatment options [5].

## Additional file

Additional file 1: **Figure S1.** Haematoxylin-eosin stainings of patients’ biopsies, as well as direct immunofluorescence staining pictures with IgA for diagnostics are shown. Both the histological and the immunofluorescence findings are similar in the 3 cases. The histology shows spongiosis and intraepidermal blisters, as well as infiltrates of neutrophilic granulocytes (hematoxylin-eosin, original magnification × 100). Direct immunofluorescence microscopy revealed IgA deposits at the upper part of the epidermis (original magnification × 200). (TIF 548 kb)

## Abbreviations

MGUS: monoclonal gammopathy of undetermined significance
RAR: retinoic acid receptor
RXR: retinoid-X-receptor
