# Supplementary figures and images for: Systemic retinoids for treatment of recalcitrant IgA pemphigus
Source: Orphanet J Rare Dis. 2018 Sep 18;13:163. doi: 10.1186/s13023-018-0899-y (PMC6145102; doi:10.1186/s13023-018-0899-y)

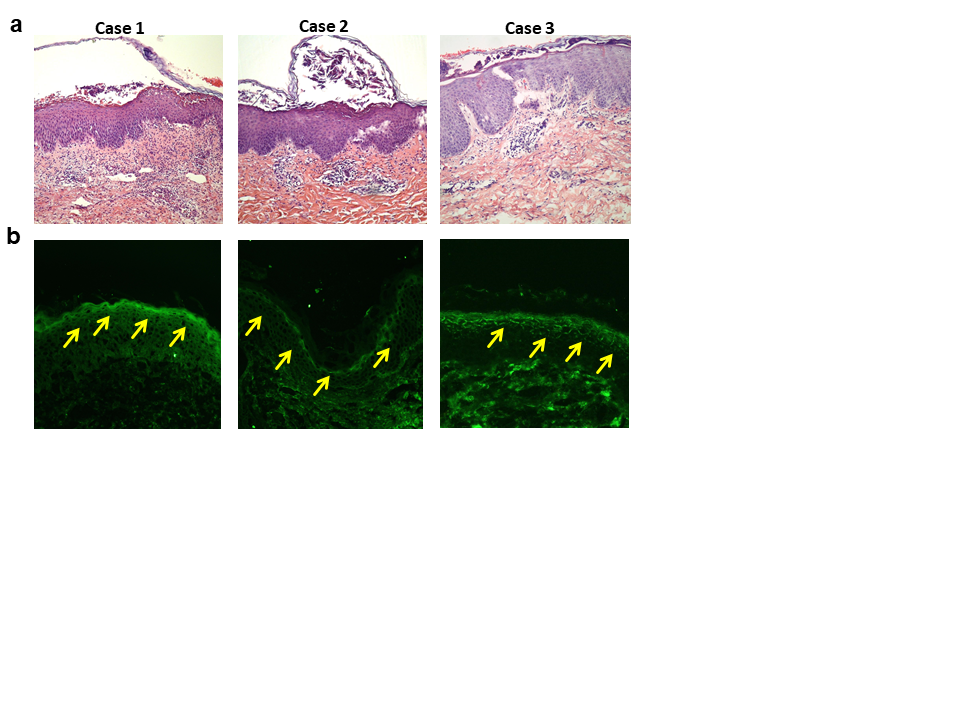

Supplement: Supplementary file 1 — Figure S1. Haematoxylin-eosin stainings of patients’ biopsies, as well as direct immunofluorescence staining pictures with IgA for diagnostics are shown. Both the histological and the immunofluorescence findings are similar in the 3 cases. The histology shows spongiosis and intraepidermal blisters, as well as infiltrates of neutrophilic granulocytes (hematoxylin-eosin, original magnification × 100). Direct immunofluorescence microscopy revealed IgA deposits at the upper part of the epidermis (original magnification × 200). (TIF 548 kb) [file 13023_2018_899_MOESM1_ESM.tif]
